# Intraoperative Pathologie bei Kopf-Hals-Tumoren

**DOI:** 10.1007/s00106-025-01715-z

**Published:** 2026-01-21

**Authors:** Alexander Quaas, Su Ir Lyu

**Affiliations:** Institut für Pathologie, Universitätsklinikum Köln, Kerpener Str. 62, 50937 Köln, Deutschland

**Keywords:** Schnellschnitt, Resektionsrand, Resektionsrand, Stimulierte Raman-Histologie, Schnelle Immunohistochemie, Frozen section, Surgical margin, Resection margin, Stimulated Raman histology, Rapid immunohistochemistry

## Abstract

Die intraoperative Pathologie ist ein zentrales Instrument während der chirurgischen Eingriffe, wo die präzise Resektionsrandbeurteilung und die Einschätzung der Dignität von Tumoren maßgeblich über den funktionellen Erhalt und die onkologische Sicherheit entscheiden. In der Otorhinolaryngologie kommt die Methode v. a. bei Plattenepithelkarzinomen („squamous cell carcinoma“, SCC) der Mundhöhle, des Pharynx und des Larynx zum Einsatz. Die diagnostische Präzision ist für SCC insgesamt hoch, variiert jedoch je nach Lokalisation und wird insbesondere durch Vorbehandlungen wie Radiotherapie oder neoadjuvante Chemotherapie eingeschränkt. Für Nicht-SCC-Entitäten, z. B. Speicheldrüsentumoren oder Lymphome, ist die Aussagekraft intraoperativer Diagnostik deutlich begrenzter. Neue Technologien wie stimulierte Raman-Histologie und schnelle Immunohistochemie könnten zukünftig die intraoperative Diagnostik im Kopf-Hals-Bereich beschleunigen und weiter präzisieren.

## Abstract (en)

Intraoperative pathology plays a central role during surgical interventions where precise margin assessment and evaluation of tumor dignity are critical for balancing functional preservation and oncological safety. In otorhinolaryngology, the method is used particularly in squamous cell carcinomas (SCC) of the oral cavity, pharynx and larynx. The diagnostic accuracy is generally high for SCC but varies depending on the anatomical site and is particularly limited by pretreatment processes, such as radiotherapy or neoadjuvant chemotherapy. For non-SCC entities, such as salivary gland tumors or lymphomas, the diagnostic value of intraoperative pathology is considerably more limited. New technologies such as stimulated Raman histology and rapid immunohistochemistry could accelerate and further refine intraoperative diagnostics in the head and neck region in the future.

## Hinführung zum Thema

Die intraoperative Pathologie hat sich als unverzichtbares Werkzeug in der HNO(Hals-Nasen-Ohren)-Onkologie etabliert. Insbesondere bei Resektionen von Tumoren im Kopf-Hals-Bereich, wo millimetergenaue Schnittränder über funktionelle Ergebnisse und Prognose entscheiden, ist die Schnellschnittdiagnostik integraler Bestandteil des operativen Vorgehens.

## Grundlagen

Die intraoperative Pathologie umfasst sämtliche diagnostische Verfahren, die während einer Operation eingesetzt werden, um dem chirurgischen Team zeitnah Informationen für das weitere Vorgehen zu liefern. Im klinischen Sprachgebrauch wird sie häufig gleichgesetzt mit der Schnellschnittdiagnostik („frozen section“). Streng genommen, ist der Schnellschnitt jedoch nur eine Methode innerhalb dieses diagnostischen Spektrums.

**Abb. 1:**
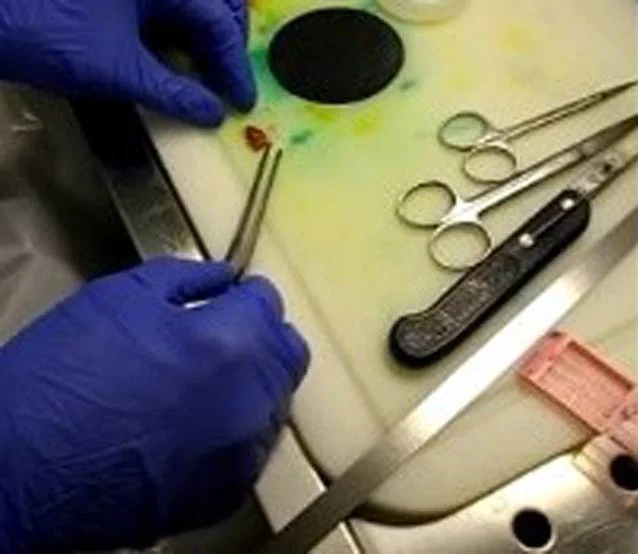
Makroskopische Begutachtung des Präparats

**Abb. 2:**
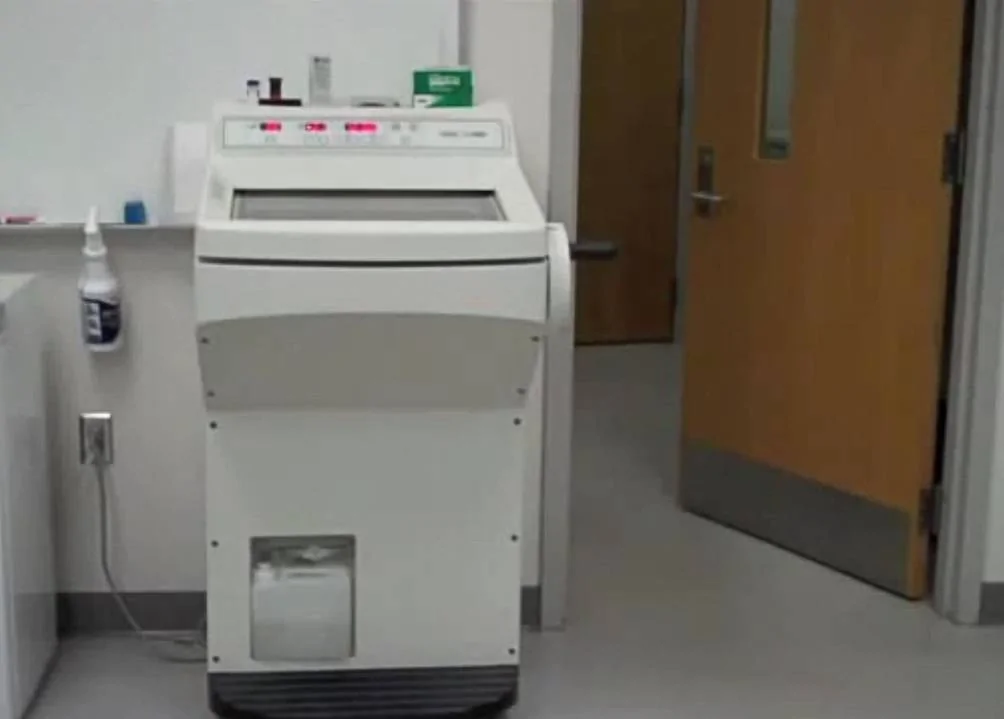
Einfrieren und Schneiden des gefrorenen Materials bei etwa −20 °C im Kryostaten

**Abb. 3:**
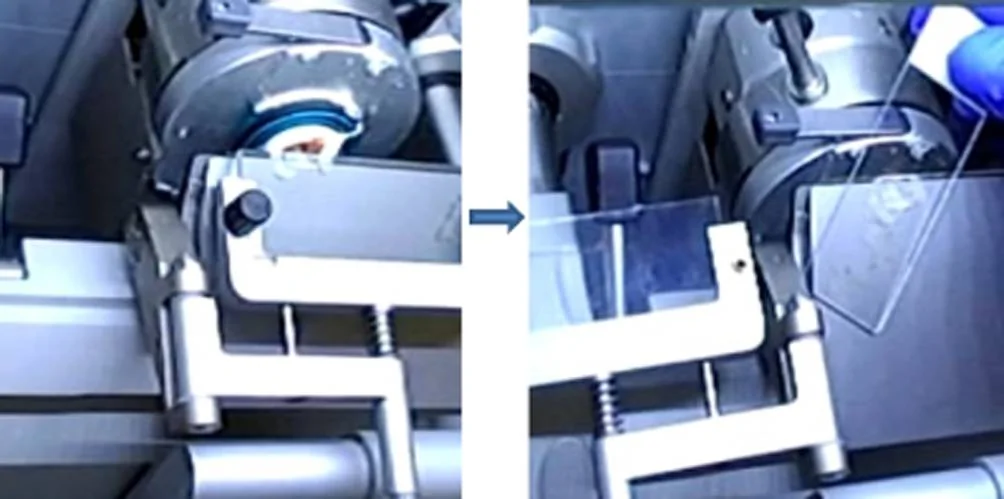
Anfertigung dünner (ca. 5 µm) Gewebeschnitte

**Abb. 4:**
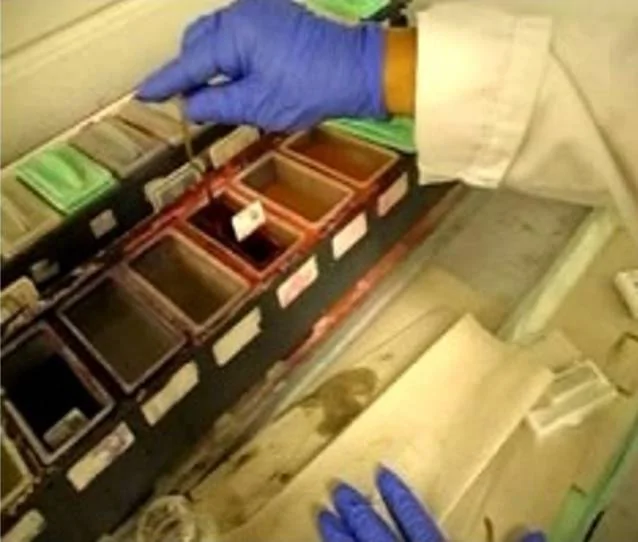
Färbung mit Hämatoxylin und Eosin (Standardfärbung)

Der Schnellschnitt stellt die häufigste und wichtigste Form intraoperativer Pathologie dar. Das Verfahren beinhaltet die makroskopische Begutachtung des Präparats (Abb. 1), das Einfrieren bei etwa −20 °C im Kryostaten (Abb. 2), die Anfertigung dünner (ca. 5 µm) histologischer Schnittpräparate (Abb. 3) sowie deren Färbung mit Hämatoxylin und Eosin (Abb. 4). Innerhalb von etwa 10 min ist diese Gewebeaufarbeitung abgeschlossen und steht zur fachärztlichen Begutachtung zur Verfügung.

Darüber hinaus gehören zur intraoperativen Pathologie auch zytologische Verfahren wie Quetsch- („squash smears“) und Abklatschpräparate („touch imprints“), die insbesondere in der Neurochirurgie Anwendung finden. Bei speziellen Fragestellungen kann zusätzlich eine schnelle Immunhistochemie („rapid immunohistochemistry“) eingesetzt werden. Diese neue und bislang wenig etablierte Methode unter Schnellschnittbedingungen, aber nicht am formalinfixierten Gewebe erlaubt die Darstellung von Proteinen auf dem Gewebeschnitt. Die Antikörperinkubationszeit beträgt etwa 10–12 min, sodass diese Schnellimmunhistochemie final interpretierbare Ergebnisse nach 20 min liefern kann. Unterscheidungen zwischen z. B. Karzinomen, malignen Melanomen und Lymphomen sind somit möglich [7]. Neue bildgebende Technologien wie die stimulierte Raman-Histologie („stimulated Raman histology“, SRH) liefern digitale, farbstofffreie Gewebebilder in quasi-histologischer Qualität und eröffnen neue Perspektiven für die intraoperative Diagnostik. Diese Technologie ermöglicht eine nahezu Echtzeitbeurteilung von unfixiertem Gewebe ohne die Notwendigkeit einer Färbung oder Kryoschnittpräparation. Der große Vorteil liegt in der schnellen digitalen Erfassung und KI(künstliche Intelligenz)-gestützten Klassifikation histologischer Strukturen, wodurch intraoperative Entscheidungen beschleunigt und objektiviert werden können. Zudem bleibt das Gewebe für weiterführende Analysen erhalten. Nachteile bestehen bislang in den hohen Geräte- und Implementierungskosten, in der begrenzten Verfügbarkeit sowie in der Notwendigkeit einer algorithmischen Schulung und Validierung der Bildauswertung [4, 8].

Gerade in der HNO-Heilkunde wird die intraoperative Pathologie nahezu synonym mit dem Schnellschnitt verwendet, da dieser die klinisch relevantesten Fragestellungen – etwa die Resektionsrandbeurteilung bei Plattenepithelkarzinomen – zuverlässig beantwortet. Gleichwohl wird der Oberbegriff zunehmend bedeutsamer, da sich die intraoperative Diagnostik kontinuierlich diversifiziert.

## Indikationen

In der HNO-Chirurgie stehen 3 Hauptindikationen im Vordergrund:

1. Resektionsrandbeurteilung (R-Status), insbesondere bei Plattenepithelkarzinomen von Mundhöhle, (Oro‑)Pharynx und Larynx, aber auch Speicheldrüsenkarzinomen;
2. Dignitätsbestimmung bei unklaren Läsionen (z. B. bei Schleimhautveränderungen, Speicheldrüsentumoren oder suspekten Lymphknoten);
3. Abklärung von Metastasen und entsprechenden Differenzialdiagnosen wie Lymphomen, insbesondere in zervikalen Lymphknoten.

Darüber hinaus wird die intraoperative Pathologie unter anderem bei viszeralchirurgischen, neurochirurgischen und orthopädischen Eingriffen eingesetzt, bleibt aber im HNO-Bereich eine der häufigsten Anwendungen [5].

## Klinische Fragestellungen

In der Kopf-Hals-Chirurgie stehen intraoperative Konsultationen im Wesentlichen im Dienst klar definierter klinischer Fragestellungen. Für den Operateur ist entscheidend, ob ein Resektionsrand tumorfrei ist, ob die Dignität einer Läsion eine unmittelbare Erweiterung der Operation erfordert und ob in einem Lymphknoten metastatische Tumoranteile oder ein Lymphom vorliegen. Diese Fragestellungen beeinflussen unmittelbar das chirurgische Vorgehen, die Ausdehnung der Resektion und potenziell die Rekonstruktionsplanung.

Zugleich bestehen aus pathologischer Sicht technische und methodische Rahmenbedingungen, die die Beantwortung dieser Fragen intraoperativ limitieren. Dazu zählen die nur begrenzte Gewebemenge, Gefrierartefakte, die eingeschränkte Verfügbarkeit der Immunhistochemie und die Notwendigkeit einer raschen Befundung. Im Vergleich zur Paraffindiagnostik ist die morphologische Interpretation daher anspruchsvoller, und die Aussagekraft variiert deutlich je nach Tumorentität und klinischem Kontext. Während Plattenepithelkarzinome der oberen Aerodigestivtrakte häufig gut intraoperativ beurteilbar sind, weisen andere Entitäten – insbesondere Speicheldrüsentumoren oder Lymphome – geringere diagnostische Sicherheit auf.

Die intraoperative Pathologie dient somit in erster Linie der schnellen Entscheidungsfindung im Operationssaal und weniger der endgültigen histopathologischen Diagnose. Dieses Spannungsfeld zwischen klinischer Erwartung und methodischen Limitationen bildet den Rahmen für die im Folgenden dargestellten Aspekte zur Präzision und zu den Herausforderungen der Schnellschnittdiagnostik.

## Präzision

Die diagnostische Präzision der intraoperativen Pathologie ist im HNO-Bereich v. a. bei Plattenepithelkarzinomen hoch, erfordert jedoch Erfahrung (z. B. Differenzialdiagnose: nekrotisierende Sialometaplasie von Ausführungsgangepithelien kleiner Speicheldrüsen nach vorangegangen Probenbiopsien kann ein Plattenepithelkarzinom vortäuschen). Für andere Entitäten wie Speicheldrüsentumoren – insbesondere für das adenoid-zystische Karzinom – oder Lymphome ist der Schnellschnitt aufgrund fehlender entitätsdefinierender Merkmale und der fehlenden Immunhistochemie hingegen nur eingeschränkt aussagekräftig und spielt intraoperativ kaum eine Rolle. Mehrere Studien belegen, dass die Genauigkeit bei der Randbeurteilung in der Mundhöhle und im Larynx bei über 90 % liegt [2]. Limitierend sind v. a. Vorbehandlungen: Nach Radiotherapie oder Chemotherapie verändert sich die Histomorphologie des Gewebes und des Tumors, wodurch die Interpretation erschwert wird. Artefakte durch Einfrieren und die stark limitierte Möglichkeit von Spezialfärbungen stellen zusätzliche Herausforderungen dar [9].

Die diagnostische Präzision variiert zudem je nach Ausprägung intraepithelialer Veränderungen. Während hochgradige Dysplasien/Carcinoma in situ im Schnellschnitt häufig zuverlässig erkannt werden können, bereitet die Abgrenzung von Low-grade-Dysplasien gegenüber reaktiven Veränderungen größere Schwierigkeiten. Die sichere Identifikation einer Mikroinvasion stellt eine besondere Herausforderung dar, da stromale Desmoplasie, Entzündung oder Kryoartefakte die Bewertung erschweren können. In vielen Fällen gelingt eine definitive Abklärung erst im Paraffinmaterial mit optimaler Schnittqualität und ggf. ergänzender Immunhistochemie.

Das orale Plattenepithelkarzinom („oral squamous cell carcinoma“, OSCC) macht rund 90 % aller malignen Neoplasien der Mundhöhle aus und ist damit eine der häufigsten Tumorentitäten im HNO-Bereich [2]. Zu den Hauptrisikofaktoren zählen Nikotin- und Alkoholkonsum sowie Infektionen mit humanen Papillomviren (HPV). HPV-assoziierte Oropharynxkarzinome weisen häufig eine scharf begrenzte Tumorinvasion ohne ausgeprägte Übergangsformen von dysplastischem zu invasivem Epithel auf [6]. Dies könnte die intraoperative Resektionsrandbeurteilung im Vergleich zu klassischen SCC mit breiteren, irregulären Tumorausläufern oder koexistierenden Schleimhautdysplasien erleichtern. Gleichzeitig wird derzeit intensiv diskutiert, ob bei HPV-positiven Tumoren ein geringerer Sicherheitsabstand (z. B. 2–3 mm statt der traditionellen 5 mm) onkologisch ausreichend sein könnte. Belastbare prospektive Daten hierzu liegen jedoch bislang nicht vor, sodass die Auswirkungen auf die intraoperative Randbewertung gegenwärtig noch unklar sind [6]. Die Prognose wird maßgeblich durch das Tumorstadium, die Invasionstiefe, das perineurale und vaskuläre Wachstum sowie den Resektionsrandstatus bestimmt [3]. Unter diesen Faktoren gilt der Resektionsrand als entscheidender unabhängiger Prognoseparameter, da positive Ränder mit einem deutlich erhöhten Lokalrezidivrisiko assoziiert sind [1].

Die Schnellschnittdiagnostik ist daher in der OSCC-Chirurgie weit verbreitet, um intraoperativ tumorfreie Ränder sicherzustellen. In einer größeren retrospektiven Studie mit über 250 OSCC-Fällen lag die Sensitivität der Schnellschnittdiagnostik bei etwa 89 %, die Spezifität bei 95 % und die Gesamttreffsicherheit bei rund 92 %. Bemerkenswert war, dass die Genauigkeit mit zunehmendem Tumorstadium und -grad anstieg [2]. Dies unterstreicht die Bedeutung des Schnellschnitts besonders in fortgeschrittenen Tumorstadien. Gleichzeitig zeigen andere Arbeiten widersprüchliche Ergebnisse hinsichtlich des Einflusses auf die Gesamtprognose: Während einige Studien einen prognostischen Vorteil durch konsequente intraoperative Randkontrolle belegen, sehen andere keinen signifikanten Überlebensvorteil [10]. Dennoch bleibt die Schnellschnittdiagnostik für die Sicherstellung tumorfreier Ränder im klinischen Alltag unverzichtbar.

## Herausforderungen

Die intraoperative Pathologie im Kopf-Hals-Bereich ist durch spezifische Herausforderungen geprägt. Neben den anatomisch-funktionellen Besonderheiten der Kopf-Hals-Region ergeben sich die intraoperativen Herausforderungen maßgeblich aus der Diskrepanz zwischen klinischer Erwartung und den technischen Möglichkeiten der Schnellschnittdiagnostik. Während der Operateur eine schnelle und möglichst eindeutige Beurteilung des Resektionsrands und der Dignität einer Läsion oder eines suspekten Lymphknotens benötigt, steht der Pathologie intraoperativ nur begrenzte Gewebequalität, eingeschränkte Immunhistochemie und eine kleine Stichprobe des tatsächlichen Resektionsrands zur Verfügung. Diese strukturelle Begrenzung bildet die Grundlage für viele der nachfolgend genannten diagnostischen Schwierigkeiten.

Ein praktischer Aspekt betrifft die Orientierung und Zuordnung der oft zahlreichen Randschnitte, wie sie insbesondere bei Resektionen der Mundhöhle und des Oropharynx entstehen. Da der Pathologie intraoperativ meist nur kleine, separate Präparate vorliegen, ist eine präzise anatomische Zuordnung nur dann möglich, wenn die Proben eindeutig orientiert und entsprechend markiert sind. Eine klare Kommunikation und Orientierungshilfen aus dem Operationssaal erleichtern daher die intraoperative Beurteilung erheblich und tragen zur diagnostischen Sicherheit bei.

Tumoren wachsen hier oft entlang anatomisch komplexer Strukturen, und funktionelle Aspekte wie Stimm- und Schluckfunktion setzen enge Grenzen für radikale Resektionen. Pathologische Artefakte durch Einfrieren sowie Therapieeffekte nach Radiochemotherapie erschweren die sichere Beurteilung [9]. Insbesondere nach neoadjuvanter oder definitiver Radiochemotherapie zeigen Kopf-Hals-Plattenepithelkarzinome häufig ein diskontinuierliches Regressionsmuster mit verstreut liegenden Residualtumornestern, die nicht mehr der klassischen kompakten Tumorfront entsprechen. Diese mikroskopisch kleinen Residualareale können im Schnellschnitt leicht übersehen werden, da nur ein begrenzter Anteil des Resektionsrands histologisch erfasst wird. Neue intraoperative Ansätze versuchen daher, Residualtumor unabhängig von einer zusammenhängenden Invasionsfront zu detektieren. Hierzu zählen bildgebende Verfahren wie die SRH mit großflächiger, farbstofffreier Oberflächenerfassung und fluoreszenzbasierte Sonden, die innerhalb weniger Minuten Tumorgewebe selektiv markieren. In Kombination mit KI-gestützten Auswertungsmethoden könnten solche Techniken zukünftig die Sensitivität der Residualtumorerkennung nach Radiochemotherapie deutlich verbessern. Jenseits der üblichen Plattenepithelkarzinome und ihrer Dysplasievorstufen erschwert eine Reihe seltener Tumoren der Mundhöhle (z. B. Speicheldrüsentumoren und maligne Melanome) die Aussagekraft von Schnellschnittanalysen. So kann es an Probebiopsien aus einem Speicheldrüsentumor, insbesondere unter Schnellschnittbedingungen, unmöglich sein, die verlässliche Entität und Dignität zu diagnostizieren. Aufgrund der enormen morphologischen Vielfalt der über 30 unterschiedlichen benignen und malignen Tumorentitäten der Speicheldrüsentumoren gilt nach wie vor der Wahlspruch, dass die Dignität erst nach Bestimmung der spezifischen Entität zu erfolgen hat. Hier gilt es möglicherweise auf die Beurteilung am Paraffinmaterial zu warten, ggf. sind auch entitätsbeweisende Zusatzuntersuchungen nötig. Die Vorläuferläsionen oder intraepithelialen Ausläufer eines malignen Melanoms wachsen unter anderem pagetoid, einzelzellig entlang der im Übrigen unauffälligen Schleimhaut und können im Schnellschnitt übersehen werden. Häufig gelingt eine verlässliche Randbeurteilung erst am Paraffinmaterial nach immunhistochemischer Aufarbeitung. Inwieweit die oben genannten Spezialtechniken (intraoperative Schnellimmunhistochemie oder SRH) hier präzisere Aussagen erlauben, muss in Studien geklärt werden, sie liefern aber vielversprechende Möglichkeiten. Eine zusätzliche Herausforderung stellt die Zeitabhängigkeit der konventionellen Schnellschnittdiagnostik dar. In dezentralen Versorgungsstrukturen oder bei größeren räumlichen Distanzen zwischen Operationssaal und Pathologie können Transport- und Prozesszeiten die intraoperative Befundübermittlung verzögern. Zudem erfordert die hohe Zahl an intraoperativen Konsultationen eine ständige Verfügbarkeit spezialisierter Pathologinnen und Pathologen.

## Ausblick

Neue Technologien könnten die intraoperative Diagnostik im HNO-Bereich entscheidend verändern. Die SRH liefert digitale, farbstofffreie Gewebebilder in Echtzeit und könnte so Artefakte reduzieren und die Diagnostik beschleunigen [8]. In Kombination mit KI eröffnet die SRH die Möglichkeit einer standardisierten und automatisierten Analyse, die insbesondere im hochfrequenten Setting der Kopf-Hals-Chirurgie von großem Nutzen wäre. Auch intraoperative Immunhistochemie gewinnt an Bedeutung, wenn Differenzialdiagnosen während der Operation rasch geklärt werden müssen. Derzeit befindet sich auch eine weitere Technik, die eine fluoreszenzbasierte Proteindarstellung von Tumorgewebe nutzt, in der Erprobung. Hier soll innerhalb von 10 min eine sichere Unterscheidung von Tumorgewebe und Normalgewebe möglich sein [7]. Sollte diese Technik reproduziert verlässliche Daten liefern, wäre sie für die Tumorrandbeurteilung eine weitere Alternative zum klassischen morphologiebasierten Schnellschnitt.

## Fazit für die Praxis

- Der intraoperative Schnellschnitt ist in der HNO(Hals-Nasen-Ohren)-Chirurgie unverzichtbar.
- Hauptindikationen sind die Resektionsrandbeurteilung, die Dignitätsbestimmung und die Lymphknotenabklärung.
- Die Präzision ist hoch, wird jedoch durch Artefakte und Vorbehandlungen eingeschränkt.
- Erfahrung und Spezialisierung der Pathologinnen und Pathologen sind entscheidend.
- Neue Technologien wie die stimulierte Raman-Histologie könnten die Diagnostik im Kopf-Hals-Bereich weiter optimieren.

## References

[1] Algadi HH, Abou-Bakr AA, Jamali OM et al (2020) Toluidine blue versus frozen section for assessment of mucosal tumor margins in oral squamous cell carcinoma. BMC Cancer 20:1147 10.1186/s12885-020-07644-0 PMC7691066 33238944

[2] Ali JP, Mallick BA, Rashid K et al (2024) Diagnostic accuracy of intraoperative frozen section for margin evaluation of oral cavity squamous cell carcinoma. BMC Res Notes 17:43 10.1186/s13104-024-06698-8 PMC10835936 38303028

[3] Chamoli A, Gosavi AS, Shirwadkar UP et al (2021) Overview of oral cavity squamous cell carcinoma: Risk factors, mechanisms, and diagnostics. Oral Oncol 121:105451 10.1016/j.oraloncology.2021.105451 34329869

[4] Hollon TC, Pandian B, Adapa AR et al (2020) Near real-time intraoperative brain tumor diagnosis using stimulated Raman histology and deep neural networks. Nat Med 26:52–58 10.1038/s41591-019-0715-9 PMC6960329 31907460

[5] Mcintosh ER, Harada S, Drwiega J et al (2015) Frozen section: guiding the hands of surgeons? Ann Diagn Pathol 19:326–329 10.1016/j.anndiagpath.2015.07.004 26320052

[6] Pool C, Weaver T, Zhu J et al (2021) Surgical Margin Determination in the Era of HPV-Positive Oropharyngeal Cancer. Laryngoscope 131:E2650–E2654 10.1002/lary.29533 PMC10797598 33797105

[7] Reinhardt D, Ter Mors B, Driessen MD et al (2024) Visually distinguishing between tumor tissue and healthy tissue within ten minutes using proteolytic probes. Sens Diagn 3:1319–1328

[8] Steybe D, Poxleitner P, Metzger MC et al (2023) Stimulated Raman histology for histological evaluation of oral squamous cell carcinoma. Clin Oral Investig 27:4705–4713 10.1007/s00784-023-05098-9 PMC10415463 37349642

[9] Taqi SA, Sami SA, Sami LB et al (2018) A review of artifacts in histopathology. J Oral Maxillofac Pathol 22:279 10.4103/jomfp.JOMFP_125_15 PMC6097380 30158787

[10] Varvares MA, Poti S, Kenyon B et al (2015) Surgical margins and primary site resection in achieving local control in oral cancer resections. Laryngoscope 125:2298–2307 10.1002/lary.25397 26011037

